# Special Issue: “Ion Channels as a Potential Target in Pharmaceutical Designs 2.0” †

**DOI:** 10.3390/ijms26178418

**Published:** 2025-08-29

**Authors:** Sheng-Nan Wu

**Affiliations:** 1 Department of Physiology, College of Medicine, National Cheng Kung University, Tainan 70101, Taiwan; snwu@ncku.edu.tw; 2 Department of Research and Education, An Nan Hospital, China Medical University, Tainan 70965, Taiwan

Ion channels embedded in the cell membrane are essential for regulating the movement of varying ions into and out of cells, thereby playing a vital role in shaping membrane potential. Changes in membrane potential—such as depolarization and hyperpolarization—activate distinct voltage-gated ion channels, each with unique gating properties and activation thresholds. These voltage-gated currents are subject to modulation by a wide range of pharmacological agents, which can alter the firing frequency and functional behavior of excitable cells.

Recent findings suggest that many drugs, beyond targeting intracellular components like the cytoplasm or nucleus, often localize to the cell membrane prior to cellular entry. This membrane residency implies potential interactions with ion channels or associated regulatory mechanisms, which may influence both the drug’s primary site of action and the electrophysiological properties of the cells. Such interactions could have profound implications for cellular excitability and overall function.

Given the growing recognition of these membrane-level dynamics, it is increasingly important to investigate how specific drugs modulate ion channel activity both directly and indirectly. Furthermore, understanding the structural characteristics of these drugs or compounds and their mechanisms of action at the membrane interface is crucial for advancing therapeutic strategies.

To foster continued exploration in this area, we are pleased to extend the original Special Issue and invite researchers to contribute their novel insights and discoveries. This platform aims to promote meaningful dialogue and collaboration in the study of ion channel pharmacology and membrane-targeted mechanisms.

A recent study by Duran-Morales et al. [1] elucidates the molecular mechanisms underlying mechanosensitive gating in the *OSCA1.2* ion channel. By integrating genetic encoding of the photoactivatable crosslinker *p*-benzoyl-L-phenylalanine (BzF), UV-induced crosslinking, Ca^2+^ imaging, and molecular dynamics simulations, the authors identified key residues—F22, H236, and R343—as critical contributors to channel gating. Crosslinking at these positions locked *OSCA1.2* in a non-conducting state, highlighting their functional importance. Of note, F22 interacts with lipids near the activation gate, suggesting a role in lipid-mediated gating. H236 lines a lipid-filled cavity, potentially influencing membrane tension sensing. R343 forms inter-subunit contacts, implicating it in allosteric communication across the channel.

Structural analyses revealed that these residues are strategically distributed across different domains, supporting a model in which mechanical gating arises from a coordinated network of interactions that allosterically converge on the activation gate. These findings advance our understanding of mechanotransduction by demonstrating how distant structural elements contribute to force sensing and gating in OSCA channels. Targeting these structural elements by specific small molecules remains to be notably determined.

A recent work by Furdak et al. [2] investigates whether the total antioxidant capacity (TAC) of complex materials—specifically an aqueous garlic extract—can be better understood by using a series of assays with oxidants/indicators of varying redox potentials (E°′) ranging from 0.11 to 1.15 V. The goal was to determine if antioxidants with different redox potentials could be selectively detected based on thermodynamic criteria.

Nine antioxidants and garlic extract were tested using nine different assays. Antioxidant activities were quantified as mol Trolox equivalents/mol compound. Some antioxidants did not react with indicators having low redox potentials, consistent with thermodynamic expectations. However, no consistent correlation was found between antioxidant activity and the redox potential of the oxidants/indicators. The garlic extract’s TAC showed no regular pattern across redox potentials, with the highest activity observed in the 2,2′-azinobis-(3-ethylbenzothiazoline-6-sulfonate) radical (ABTS^•^) assay. These results suggest that kinetic factors, rather than thermodynamic ones, primarily govern antioxidant activity and TAC measurements in these assays [2].

A recent study by Butiulca et al. [3] investigated the pharmacokinetics of ropivacaine and its active metabolite, 3-OH ropivacaine, after an ultrasound-guided PECS II block. A PECS II block is a regional anesthesia technique performed under ultrasound guidance, primarily used for post-operative pain management following breast and axillary surgeries. The goal was to understand how these compounds behave in the body over time following interpectoral administration, as these data were previously limited. In this study [3], researchers collected venous blood samples from 18 patients at specific intervals (0, 1, 3, 6, and 24 h) after they received a PECS II block with a ropivacaine-lidocaine mixture. They used a validated LC-MS/MS method to quantify the plasma concentrations.

The study [3] found that ropivacaine reached a peak plasma concentration (C_max_) of 167.5 ± 28.3 ng/mL at a peak time (T_max_) of 1.3 ± 0.2 h. Its metabolite, 3-OH-ropivacaine, peaked at 124.1 ± 21.4 ng/mL at 2.3 ± 0.3 h. Notably, both the drug and its metabolite showed prolonged systemic exposure, with half-lives of 19.4 ± 2.8 h and 29.2 ± 3.1 h, respectively. The findings demonstrate that the PECS II block leads to sustained systemic levels of both ropivacaine and its primary metabolite.

A current study by Orea et al. [4] demonstrates that pharmacological preconditioning (PPC) with the mitochondrial ATP-sensitive K^+^-channel (mitoK_ATP_) opener diazoxide (DZX) enhances heart rate by upregulating *HCN4* channel expression in sinoatrial (SA) nodal cells. DZX increased *HCN4* currents, mRNA, and protein levels, as well as immunofluorescence signals, effects that were abolished by the mitoK_ATP_ inhibitor 5-hydroxydecanoate and the reactive oxygen species (ROS) scavenger N-acetylcysteine. Mechanically, PPC promoted nuclear translocation of c-Fos and reduced REST/NRSF abundance, implicating these transcriptional regulators in *HCN4* upregulation. These findings reveal a novel mechanism by which PPC modulates pacemaker activity via ROS-dependent transcriptional control of *HCN4* channels. However, whether other types of ionic channels in SA node can be transcriptionally regulated by PPC remains to be further investigated.

Despite significant progress in cancer therapies, therapeutic resistance and relapse—often driven by cancer stem cells (CSCs)—remain major challenges. CSCs are rare, therapy-resistant cells capable of regenerating tumors, necessitating deeper understanding of their survival mechanisms. Emerging evidence reveals that CSCs express ion channels typically found in excitable tissues, which play crucial roles beyond electrophysiology by regulating CSC fate and function.

In this review [5], the researchers examine the wide array of ion channels expressed in CSCs, shedding light on the underlying mechanisms by which these channels regulate CSC behavior, cellular dynamics, and survival. Ion channels were also emphasized as promising diagnostic markers and therapeutic targets, offering novel strategies to disrupt CSC-driven tumor progression. The findings identify key gaps in current knowledge and outline priorities for future research. By focusing on ion channel-mediated regulation, the review underscores a compelling and underexplored avenue for advancing cancer treatment through CSC-targeted interventions.

Oligodendrocyte precursor cells (OPCs) are a dynamic glial population with lifelong proliferative and migratory abilities. Traditionally known for differentiating into oligodendrocytes (OLs) and forming myelin to support neuronal conduction, OPCs are now recognized for their non-canonical roles, including direct interactions with neurons and contributions to central nervous system (CNS) homeostasis.

A defining characteristic of OPCs is their expression of diverse ion channels, which regulate key cellular processes, such as proliferation, migration, and differentiation. Given their widespread presence across the CNS, OPCs are increasingly implicated in the pathogenesis of various neurological disorders. Another review article by Wang et al. [6] provides a detailed overview of the physiological and pathological roles of ion channels in OPCs, their involvement in CNS dysfunction and disease progression, and the therapeutic potential of targeting OPC ion channels in conditions, such as multiple sclerosis, spinal cord injury, amyotrophic lateral sclerosis, psychiatric disorders, Alzheimer’s disease, and neuropathic pain. The article [6] also explores emerging therapeutic strategies aimed at modulating OPC ion-channel activity, offering promising avenues for future CNS disease treatments.

A review paper by Lu et al. [7] focuses on the K_V_7.2, K_V_7.3, and K_V_7.5 K^+^ channels, encoded by the *KCNQ2*, *KCNQ3*, and *KCNQ5* genes, which are widely expressed in various cell types and are critical for generating the M-type K^+^ current (*I*_K(M)_). *I*_K(M)_ is notable for its low-voltage activation threshold and slow gating kinetics, both of which play a pivotal role in regulating membrane excitability. Modulation of *I*_K(M)_ has gained increasing recognition as a potential therapeutic approach for treating a variety of neurological disorders associated with either excessive neuronal activity of dysfunctional autonomic control.

A key feature of *I*_K(M)_ is its voltage-dependent hysteresis, especially evident during isosceles-triangular ramp pulses, which reflects its complex gating behavior [7,8]. This review [7] systematically classifies small-molecule modulators of *I*_K(M)_ into two distinct categories: inhibitors, such as bisoprolol, brivaracetam, cannabidiol, nalbuphine, phenobarbital, and remdesivir; and enhancers, including flupirtine, kynurenic acid, naringenin, Q5-58, and solifenacin.

Because ion-channel proteins are predominantly located on the cell surface membrane—rather than confined within the cytoplasm or nucleus—they are more readily accessible to externally administered compounds. Proteins encoded by the *KCNQx* gene family have been implicated in a range of pathological conditions, including epileptic disorders. Targeting these channels with specific compounds is expected to modulate *I*_K(M)_ amplitude, alter gating kinetics, and influence hysteresis behavior. Such effects may provide valuable insights into the therapeutic potential of these agents for treating diseases associated with gain-of loss-of function mutations in K_V_7x/*KCNQx* channels.

Another paper by Chen et al. [9] proposes a novel mechanism by which large-conductance Ca^2+^-activated K^+^ (BK_Ca_) channels, despite their absence in mature cardiomyocytes, could influence the heart’s electrical activity. The core argument is that these channels, found in cardiac fibroblasts, become more prevalent in conditions like aging, myocardial infarction, and atrial fibrillation. The review posits that the high conductance of these BK_Ca_ channels, combined with electrical coupling between fibroblasts and adjacent cardiomyocytes, could leak to random depolarizations and increased excitability of cardiac cells. This, in turn, may contribute to the development of cardiac arrhythmias. This paper [9] also leads us to suggest that substances which affect BK_Ca_-channel activity in fibroblasts might impact cardiac function.

A notable aspect of this paper [9] is its innovative, thought-provoking hypothesis on the origins of cardiac arrhythmias. Instead of focusing solely on the well-understood ion channels in cardiomyocytes, the research highlights the often-overlooked role of non-myocyte cells, specifically cardiac fibroblasts. This approach is valuable because it may broaden the scope of potential therapeutic targets. By connecting the increased prevalence of fibroblasts in disease states to a specific ion channel mechanism, the paper provides a compelling framework for future research. This could lead to a deeper understanding of how cellular interactions beyond the classic myocyte-myocyte coupling contribute to complex heart conditions.
